# Lumbar Epidural Steroid Injections for Chronic Spinal Pain: A Clinical Review of Efficacy and Evidence

**DOI:** 10.7759/cureus.98348

**Published:** 2025-12-02

**Authors:** Amol Soin, Mayank Gupta, Alaa A Abd-Elsayed, Sara Nashi, Alex Escobar, Dustin Reynolds, Gabrielle C Montes, Richard J Witte, Evan Peskin

**Affiliations:** 1 Ohio Pain Clinic, Wright State University, Dayton, USA; 2 Department of Pain Medicine, Kansas Pain Management, Overland Park, USA; 3 Department of Anesthesiology, University of Wisconsin, Madison, USA; 4 Department of Pain Management, TriHealth, West Chester, USA; 5 Department of Anesthesiology and Pain Management, University of Toledo, Toledo, USA; 6 Department of Pain Management, OrthoNeuro, Westerville, USA; 7 Department of Anesthesiology, Wright State University Boonshoft School of Medicine, Dayton, USA; 8 Department of Anesthesiology, Wright State University, Dayton, USA; 9 Department of Chronic Pain, Insight Pain Management, Flint, USA

**Keywords:** chronic lumbar pain, lumbar epidural steroid injection, pain management, radiculopathy, spinal stenosis, steroid complications

## Abstract

Radicular pain and spinal stenosis are types of chronic lumbar spinal pain that contribute to major disability across the globe. Lumbar epidural steroid injections (LESIs) have been a primary intervention in these cases to reduce inflammation, relieve compressed nerve roots, and alleviate symptoms to avoid surgical treatments. Therefore, this review critically reviews and synthesizes up-to-date evidence on the safety, efficacy, and clinical usage of LESIs to manage chronic lumbar spinal pain, focusing especially on outcomes of different techniques and long-term effectiveness of LESIs. This study is a narrative clinical review evaluating high-quality literature from the year 2020 to 2025, focusing on academic medical centers for spinal care, orthopedic spine practices, and pain management clinics for outpatient interventions. The databases selected to collect literature for this comprehensive study were MEDLINE and PubMed, and the timeline was set from 2020 to 2025. Selected studies included cohort studies, randomized controlled trials, meta-analyses, and systematic reviews, which focused on the evaluation of the safety and efficacy of all types of LESIs. Data synthesis was qualitative and based on patients' medical condition (spinal stenosis, radiculopathy), LESI technique, short- and long-term results, and reported side effects. The majority of the studies reported impressive short-term results of LESIs, i.e., within four to 12 weeks of injections, in terms of pain relief and improved functionality, especially for transforaminal injections administered for lumbar radiculopathy. In lumbar spinal stenosis (LSS) cases, LESIs showed variable efficacy with slightly better short-term results. Literature showed inconsistency in long-term outcomes, i.e., over six months, and many cases required multiple injections. In terms of side effects, non-particulate steroids were the least problematic, with fewer serious complications in comparison. Overall, LESIs were found to be better as an effective and safe bridge therapy in many cases, but they could not be qualified as a curative option for most patients. Variability in injection techniques, heterogeneous study designs, and inconsistent criteria of patient selection restricted direct comparison of the selected studies. The majority of the studies were found in favor of short-term or intermediate-term benefits of LESIs, whereas very limited data were available on the long-term efficacy or optimal frequency of injections. LESIs are indeed effective for short-term benefits in lumbar radiculopathy and some spinal stenosis cases. Optimal outcomes depend on the LESI technique and patient stratification. Cumulative steroid risks need to be considered in case of the requirement for repeat injections. Evidence supports LESIs as being significantly more cost-effective than surgical interventions, and through quality-adjusted life years (QALYs) gained per intervention, LESIs may be the most cost-effective method for controlling early-stage LSS. LESIs should be integrated into a broader multimodal strategy to manage patients with LSS and lumbar radiculopathy.

## Introduction and background

Chronic lumbar spinal pain affects millions worldwide and significantly reduces the quality of a patient’s life while increasing the economic burden on the country’s healthcare system [1,2]. Currently, mainstay treatments for lumbar spinal pain include combinations of pharmacologic, non-pharmacologic, physical rehabilitation, and interventional treatments. Among other treatment options for lumbar spinal pain, lumbar epidural steroid injections (LESIs) are most commonly and widely used to manage radicular pain and lumbar spinal stenosis (LSS) [3,4]. The common causes of radicular pain are herniated discs and nerve compression, which ultimately lead to significant disability and reduction in quality of life [5-7]. Similarly, LSS is also characterized by a narrowed spinal canal, which leads to neurogenic claudication and chronic pain [8,9]. With LESIs, corticosteroids are directly inserted into the epidural space, which reduces inflammation and soothes nerve root irritation, thus relieving symptoms [3,4,10].

While LESIs are the primary and most common choice of treatment for patients with chronic lumbar spinal pain or to avoid surgical options, questions remain regarding their long-term efficacy [3,11,12]. Several meta-analyses and systematic reviews report the positive role of LESIs for reducing pain and improving function in short-term treatments. Studies show that LESIs result in significant pain relief for about three months for patients suffering from lumbar radiculopathy caused by disc herniation [13-16]. However, there is conflicting evidence, especially regarding the long-term effects, with studies reporting reduced effects over time, which required repeated injections [17-20]. Comparative analyses of LESIs and conventional treatments like physical therapy showed that even though the injections were quick to relieve pain, their long-term functional outcomes were not necessarily better. This has caused variation in clinical guidelines and patient selection criteria [21-23]. LESIs have shown moderate efficacy in LSS patients, especially regarding pain improvement and mobility. Clinical guidelines recommend LESIs instead of directly going for surgical options, even though their efficacy is variable based on the severity of the disease [24-26]. Despite being relatively safe, LESIs still have associated risks such as infection, nerve damage, and dural puncture, which could lead to post-dural headaches [27,28]. Considering the growing clinical interest in LESIs for LSS and radicular pain, their long-term safety and efficacy need to be critically evaluated. This clinical review aims to summarize and evaluate the current evidence supporting the use of LESIs for chronic lumbar spinal pain, and the criticisms on their long-term use, while also offering comparative analysis against alternative treatments. This review will provide a response to the recent skepticism concerning the use of LESIs for LSS, stressing the significance of appropriately selected sample populations. This review aims to explore their role for optimal pain management in clinical practice.

Mechanism of action for LESIs

The efficacy of LESIs is mainly due to the anti-inflammatory properties of the corticosteroids used in them, which influence the inflammatory pathways, as well as reduce nerve root irritation, leading to pain relief. The steroids themselves reduce pro-inflammatory cytokines, including tumor necrosis factor-alpha (TNF-α), IL-1, and IL-6, thereby modifying neural inflammation and pain sensitization. The inhibition of phospholipase A2 stops the production of prostaglandins. This leads to a reduction in local edema. Steroids offer a decrease in vascular permeability, which relieves the nerve root swelling from compression. Steroids may also stabilize the membranes of inflamed neuronal cells and glial cells, reducing ectopic nerve firing in radiculopathy. When thinking of chronic pain, there is evidence that suggests steroids directly inhibit C-fiber transmission that contributes to chronic pain [29]. Another reason for LESIs’ efficacy is the administration technique [3,4,10]. Various administration methods are available based on pathology and the patient’s anatomical situations, such as transforaminal, interlaminar, and caudal administration.

Mitigation of nerve root irritation and inflammation

Reduction of inflammation in the epidural space is the main mechanism of action for the therapeutic effect of LESIs [3,4,10]. Conditions such as a thickened ligamentum flavum, osteophytes, or herniated intervertebral discs can cause nerve root compression leading to chronic lumbar pain, especially in radiculopathy and spinal stenosis. As a result, pro-inflammatory mediators such as phospholipase A2, cytokines (e.g., interleukins and TNF-α), and prostaglandins are released, which can sensitize nociceptors and cause pain [4,29,30]. LESIs improve local edema around the affected nerve roots by reducing vascular permeability, minimizing fluid accumulation, and relieving mechanical pressure on the affected nerve roots [3,31,32]. These processes allow symptomatic relief as well as improve the quality of the patient’s life.

Multiple injection techniques

LESIs are primarily administered via three approaches: (i) transforaminal epidural steroid injection (TFESI), (ii) interlaminar epidural steroid injection (ILESI), and (iii) caudal epidural steroid injection (CESI). The selection of the administration approach for LESIs depends upon the underlying pathology, anatomical features, and the expertise of the physician.

TFESI

In the TFESI approach, corticosteroids are precisely deposited in the neural foramen to target the inflamed area near the compressed nerve root. Literature shows that this precise delivery makes the TFESI approach superior to all other approaches because it offers highly targeted and accurate delivery of the drugs to the affected area [33-35]. Despite the benefits TFESI has to offer, it poses a risk of vascular penetration, and if a case of inadvertent arterial penetration arises, it can even lead to spinal cord infarction. For such a risk, physicians recommend fluoroscopic guidance and non-particulate steroids like dexamethasone [36,37]. As such, patients may be better candidates for the TFESI approach if their pain is unilateral in nature.

ILESI

In the ILESI approach, the steroid solution is placed in the posterior space between the laminae of the adjacent vertebrae for broader drug distribution along the levels of the spine. It is commonly used for patients with central canal stenosis and diffuse lumbar pain [38-40]. Even though ILESI is not as precise as TFESI, it has a lower risk of vascular penetration. To improve the accuracy of ILESI, fluoroscopy or ultrasound is used to reduce the risk of complications [40-42]. Patients suffering from multiple-level degenerative disc disease, central canal stenosis, or bilateral symptoms are recommended to undergo ILESI as it can be more beneficial to them than TFESIs [38,40].

CESI

In the CESI approach, corticosteroids are spread throughout the epidural space by injecting through the sacral hiatus. Patients with failed back surgery syndrome (post-surgical syndrome), multiple-level stenosis, or who have anatomical limitations that do not allow them to tolerate targeted approaches can benefit from CESI [43,44]. It is a less localized approach to drug delivery than the other two, but it is the safest and easiest to administer, especially for patients with significant spinal degeneration or those in later stages of life. However, the diffuse spread of drugs in CESI requires higher volumes of injectate [45-50].

Each approach has its benefits and relative risks. Understanding how they work is critical to optimizing patient selection and maximizing therapeutic outcomes with minimal risks.

## Review

Materials and methods

This evidence-based clinical review follows the Preferred Reporting Items for Systematic Reviews and Meta-Analyses (PRISMA) guidelines [17] where applicable, but is intended to cover a broader scope than a simple systematic review.

Literature Search Strategy

**Figure 1:**
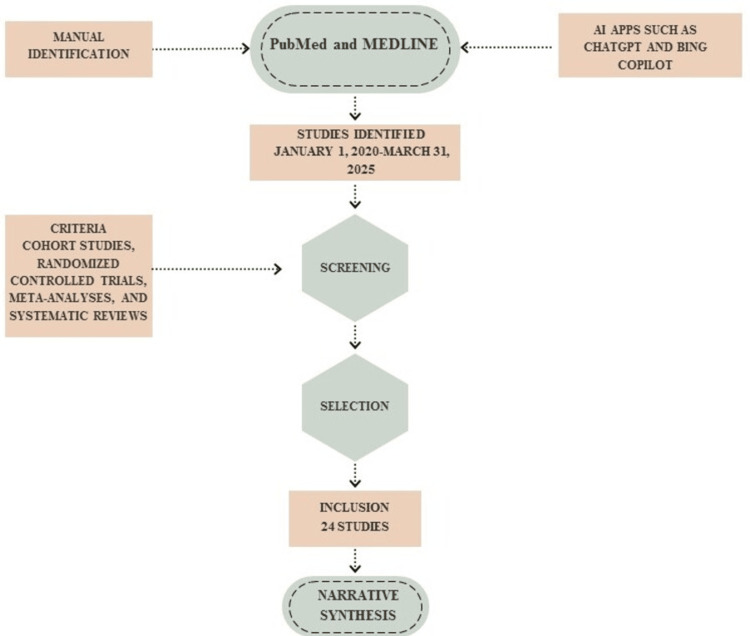
Studies selection process.

PubMed and MEDLINE databases were utilized to conduct a comprehensive literature search, and relevant studies from January 1, 2020, to March 31, 2025, were identified. AI apps such as ChatGPT and Bing Copilot were also used to supplement this search for up-to-date peer-reviewed studies. In addition, bibliographies from relevant articles and previous analyses were manually collected (Figure 1). A combination of keywords and MeSH terms was used in this study, such as LESI, lumbar spinal stenosis, lumbar epidural steroid injection, injection efficacy, long-term outcomes, lumbar radiculopathy, caudal epidural injection, transforaminal epidural steroid injection, repeated injections, interlaminar epidural injection, and pain management. The search was refined and expanded by using Boolean operators OR, AND, and NOT.

Inclusion/Exclusion Criteria

Studies were included in this review if they fulfilled the set criteria. Studies included must be published in 2020 or later. Studies selected for the review must be meta-analyses, systematic reviews, prospective and retrospective cohort studies, and randomized controlled trials (RCTs). Studies included must evaluate the safety, efficacy, pain relief duration, and results of LESIs. Studies included must have adult patients (aged 18 years or older) diagnosed with spinal stenosis or lumbar radiculopathy. Studies that compared different LESI techniques or compared LESIs with surgical or conservative treatments were also included.

Studies were excluded if they did not fulfil the requirements set for the study. Narrative reviews without any new data, editorials, and case reports were excluded. Studies that only focused on thoracic or cervical injections were excluded. Studies not available in English were excluded. Studies on pediatric populations were excluded. Studies that did not have an adequate methodology or those with outcomes irrelevant to the scope of this review were also excluded.

Study Selection

The selected studies were screened for relevance by two independent reviewers. Final inclusion was decided based on the review of the full texts of the selected studies. A third reviewer was responsible for consultation to resolve any discrepancies between the two reviewers. The finalized list included papers containing the most up-to-date and reliable studies on LESIs for lumbar spinal pathology.

Data Extraction

Studies included in this review were screened for information of choice, and the data collected from them are presented in Table 1. A template was used to standardize the collection of data for consistency. Patient outcomes were categorized based on pathology (spinal stenosis vs. radiculopathy), type of LESI (caudal, interlaminar, or transforaminal), and duration of follow-up, categorized as less than three months (short-term), three to six months (intermediate), and more than six months (long-term). The description of the data collected for this study is given in Table 1.

**Table 1:** Summary of data extracted from the included studies. LESIs: lumbar epidural steroid injections.

Data extracted	Description
Publication details	Authors, year, journal
Design	Study design and level of evidence
Population	Patient population and inclusion criteria
LESIs	Type of LESI used (caudal, interlaminar, or transforaminal)
Corticosteroids	Examples: Triamcinolone, methylprednisolone, dexamethasone
Frequency	Frequency and number of injections
Outcome	Primary and secondary measures of outcome
Follow-up	Duration of follow-up

Data Synthesis

A narrative synthesis was performed, and all data were presented in a tabular form.

Results

Twenty-four studies were selected for this review and evaluated for clinical efficacy of LESIs using different techniques and across different patient populations.

Efficacy of LESIs in Radicular Pain

For patients with radicular pain, either because of nerve root compression or a herniated disc, the short-term efficacy of LESIs was affirmed in multiple studies. Agrawal et al. (2024) found significantly reduced pain intensity in their prospective observational study [51]. They used the Numerical Rating Scale (NRS) for pain measurement and recorded the scores at 6.81 for pre-injection to 3.45 right after the injection. The benefits sustained for 12 weeks, with only a slight increase at 24 weeks to 4.23. A significant improvement was observed in the Oswestry Disability Index (ODI) score, which went from 44.19 to 29.16 after the injection, and this improvement was sustained over 24 weeks. Although patient satisfaction stopped improving over time, this suggests a reduction in benefits over a longer period [51]. In another study by Dhandapani et al. (2023), 82.69% of the patients were treated with transforaminal LESIs, and in six months, their pain was sustained, and they reported functional improvement [52]. On the other hand, 17.3% of the patients in this study needed surgery or supplementary conservative management, which highlights the variance in the responses of patients depending on factors like disc pathology subtype [52]. Table 2 highlights the comparison of outcomes of different LESI techniques for LSS and radiculopathy.

**Table 2:** Comparative summary of differences in key findings for LESIs against radiculopathy and spinal stenosis. LESIs: lumbar epidural steroid injections; NRS: Numerical Rating Scale; PT: physical therapy; US: ultrasound.

Study	Pathology	LESI technique	Short-term relief (≤3 months)	Long-term relief (>6 months)	Notes
Agrawal et al. [51]	Radiculopathy	Transforaminal	Significant (NRS reduced to 3.3)	Moderate	24-week follow-up
Gagliardi et al. [53]	Spinal stenosis	Caudal	Sustained	Not evaluated	US-guided, no side effects
Olguner et al. [54]	Foraminal stenosis	Transforaminal	Moderate	33.9% after 1 year	Best response in the foraminal subtype
Verheijen et al. [55]	Radiculopathy	Transforaminal	70% relief	44% in 16 weeks	59% needed a repeat injection
Goyal et al. [56]	Both	Caudal	Effective	Need PT/repeats	Long-term success with adjunct therapy

Efficacy of LESIs in LSS

There is inconsistency in reports of the long-term benefits of the LESIs for LSS; however, significant literature is available supporting the short-term benefits of LESIs for reducing pain and improving function. Olguner et al. (2020) found that 33.9% of patients with foraminal stenosis reported improved outcomes in 12 months, whereas those with central stenosis and herniated discs showed poorer outcomes [54]. Gagliardi et al. (2024) found significant pain relief and disability improvement in patients with LSS with caudal LESIs administered with ultrasound guidance, and the pain relief lasted for at least three months without any side effects [53]. In another study by Viswanathan et al. (2020), it was found that moderate stenosis showed better outcomes in patients treated with LESIs compared to those suffering from severely compromised central canal [57]. They also reported that the outcomes were significantly influenced by the duration of symptoms and compression severity [57]. A study by Akram et al. (2021) indicated that lumbar LESIs are more effective for spinal stenosis than caudal LESIs, with their results showing ≥50% pain reduction in 89.33% of patients after three months [58]. Despite LESIs being more effective in LSS than in radiculopathy, short-term benefits reported across the literature validate their use as a primary option before opting for surgical interventions.

Injection Technique Matters

El-Ghait et al. (2021) reported that after four weeks of treatment, transforaminal administration of LESIs showed significantly better results in pain reduction (visual analog scale, VAS) [16] and functional status (ODI) as compared to the results from interlaminar injections [59]. Group 1 (interlaminar group) showed a 52% reduction in VAS scores and a 31.13% improvement in ODI scores (p < 0.026), whereas Group 2 (transforaminal group) showed a remarkable 64.66% and 39.66% score, respectively, highlighting the difference in outcomes based on techniques [55]. Viswanathan et al. (2020) highlighted in their study that a more targeted delivery is possible with transforaminal LESIs, which allows better and superior results compared to the other LESI techniques. They reported a success rate of 76-88% for transforaminal LESIs and highlighted the significance of clinical variables, including symptom severity and duration, that can influence patient outcomes [57]. In support of caudal LESIs, Gagliardi et al. (2024) found a significant reduction in pain that lasted for three months for patients administered injections under ultrasound guidance. No notable side effects were reported, which suggested that caudal injections can be particularly useful for diffuse or multi-level pathologies [53]. A summarized comparison of the different LESI techniques is presented in Table 3, where short-term efficacy is defined by 50% or more pain reduction (VAS) or 30% or more ODI score improvement within a period of six to 12 weeks. Meanwhile, long-term efficacy means no injection repeats or surgical interventions were required after six months to sustain favorable outcomes.

**Table 3:** Comparative summary of the effectiveness and indication of different LESI techniques. LESI: lumbar epidural steroid injection; FBSS: failed back surgery syndrome.

Technique	Best suited for	Target area	Precision level	Short-term efficacy	Long-term efficacy	Safety profile	Reference
Caudal	FBSS, multiple-level stenosis, elderly	Sacral hiatus to lumbar	Low	Good (60-75%)	Often requires repeats	Low precision, high safety	[53,56,60,61]
Interlaminar	Central canal stenosis, bilateral	Posterior epidural space	Moderate	Good (50-75%)	Limited (25-40%)	Lower neurovascular risk	[62-65]
Transforaminal	Foraminal stenosis, radiculopathy	Nerve root area	High	Excellent (70-90%)	Variable (40-65%)	Moderate risk with particulates	[51,52,66,67]

Short-Term Versus Long-Term Outcomes

Literature on all LESI techniques and indications demonstrates persistently favorable short-term outcomes. Studies by El-Ghait et al. (2021) and Agrawal et al. (2024) report significant improvement in disability and pain reduction scores in four to 12 weeks post-injection [51,59]. However, these improvements plateau or decline over time. Another study by Verheijen et al. (2021) reported short-term relief in 70% of the patients who received LESI once. Among these patients, only 44% showed persistence in relief after 16 weeks, while 59% needed repeat injections [55]. Similarly, another study by Goyal et al. (2021) found evidence supporting short-term relief without long-term benefit. They found 20% of patients needed a second shot of caudal LESIs after one month, and 91% of patients in their study were given repeat injections or supplementary physical therapy to maintain relief after three months [56].

Safety and Side Effects

Despite the consensus on the safety of LESIs, multiple studies have highlighted potential adverse effects to be cautious about, especially with particulate corticosteroids and their risk of vascular occlusion compared to non-particulate ones [64-67]. Cohen et al. (2021) found that dexamethasone and other non-particulate steroids were safer but provided relief for a shorter duration compared to transforaminally administered particulate steroids, which came with rare but possible side effects [32]. Similarly, another study on non-particulate steroids by Moreira et al. (2024) compared betamethasone, methylprednisolone, and dexamethasone. They found no reported side effects and significantly less requirement for repeated injections [68]. Other systematic adverse effects have also been studied in various papers. For instance, Clare et al. (2022) found reduced markers for bone formation in 12 weeks after LESI was administered in postmenopausal women [69]. Similarly, another study by Chen et al. (2023) reported an increased risk of osteoporosis in patients with lumbar spondylosis post-LESI [70]. A summary of multiple studies focused on the safety and side effects of LESIs is presented in Table 4.

**Table 4:** Summary of safety concerns reported for LESIs across various studies. LESIs: lumbar epidural steroid injections; IOP: intraocular pressure; CSF: cerebrospinal fluid.

Type of steroid	Route of administration	Reported side effects	Notes	Ref.
Particulate/non-particulate	All three	Rare neuro events	FDA guidance noted	[18]
Particulate vs. non-particulate	Transforaminal	None. Non-particulate treatment required fewer repeats	Dexamethasone is safer	[68]
Triamcinolone	Lumbar	Reduced bone markers after 12 weeks	Postmenopausal women face the risk of osteopenia	[69]
Mixed	Lumbar	Increased risk of osteoporosis (HR = 1.23)	Based on the population cohort	[70]
Triamcinolone	Caudal	Transient increase in IOP. Resolved in 2 weeks	No loss of visual acuity	[71]
Particulate	Transforaminal	Minor side effects: vasovagal (2.4-9.6%). Rare side effects: cord infarction	Mostly minor side effects	[36]
Not specified	Lumbar pre-operation	Increased CSF leaks if the injection is given for less than 30 days before the surgery	Time-sensitive risks	[72]
Particulate and non-particulate	All	Serious spinal side effects risk of 8.1 in a million	The rate of side effects is similar for all types of steroids	[73]
Not specified	Lumbar	Increased risk of dural tears if LESI is administered within 3 months of surgery	OR = 2.9 for tear	[74]
Not specified	Mixed lumbar	Minor side effects in 1.4% of the population, with vasovagal being the most common	No major complications seen in 4209 patients	[75,76]

Discussion

Interpretation of Results

The available evidence collected by the current study confirms the hypothesis that LESIs are one of the pillars in the management of chronic lumbar radicular pain and a few cases of LSS. Their primary benefit is a temporary reduction in symptoms, functional turnover, and analgesic addiction. These effects have already been established several times in several randomized and observational studies [51,59]. Most of the patients experienced an improvement in the level of pain between four and 12 weeks following an injection of TFESIs, which is the most successful in patients with unilateral disc herniation or foraminal stenosis [53-55]. The pathophysiologic process that can contribute to these outcomes is that corticosteroids inhibit proinflammatory cytokines (e.g., TNF-α and IL-6), nerve root edema, and the activity of phospholipase A2 at the area of compression, which subsequently reduces nociceptive sensitization [29]. This process describes why acute or subacute radiculopathy has significant advantages as opposed to chronic degenerative pain, where inflammatory mediators are not overwhelming. In the case of LSS, there is variation in the results, and that depends on the type of the disease (foraminal or central), level of compression, and the chronicity of the symptoms. Research has shown that caudal and interlaminar injection will lead to moderate pain relief, particularly in mild-to-moderate stenosis, and when used together with organized physical therapy by patients [73,74]. But the long-term effects after six months are not consistent, since the control of inflammation without mechanical decompression may not be sufficient to prevent the reappearance of the symptoms. Recurrent injections thus represent a regular practice, and long-term responders tend to constitute a separate group with inflammatory but not structural stenosis [75]. Also, the literature has indicated placebo and contextual effects related to interventional procedures on pain. Perceived benefit relies on patient expectation, communication between physicians, and multidisciplinary follow-up, highlighting that LESIs cannot be considered only as a pharmacologic intervention but rather a component of a comprehensive pain management program.

Comparison With Alternative Treatments

LESIs are considered somewhere between conservative therapy and surgery. LESIs are more effective in the short term than physical therapy, nonsteroidal anti-inflammatory drugs (NSAIDs), or oral corticosteroids in terms of pain relief and early mobilization, but are not different in terms of long-term outcomes. Though LESIs could help in delaying surgery processes in the short run, the surgery must be performed eventually [76]. Recent biologic alternatives are platelet-rich plasma (PRP) injections. Initial research indicates that PRP could provide similar analgesia with reduced systemic danger, presumably due to the anti-inflammatory action of growth factors. In other cases, instead of recurrent LESIs, the minimally invasive lumbar decompression (MILD®) procedure can be a better and preferable option for improved and quick definitive care [77]. Patients would consider the cost difference in terms of risks and benefits. A cross-sectional comparison of MILD® procedure, epidural steroid injection (ESI), and laminectomy revealed the cost-effectiveness in a sample of patients with LSS and moderate-severe symptoms through a decision-analytic model with the Medicare perspective [1,2]. However, this analysis proved that the MILD® procedure was the most cost-effective among the three choices; the second most effective choice was the ESI ($37,758/quality-adjusted life years (QALY)), which used to be more effective than laminectomy ($125,985). ESI is the most cost-effective method of controlling early-stage LSS that occurs at six or fewer injections in a two-year time frame when compared to the MILD® procedure due to the fact that QALY earned is greater in the former as compared to the latter [1,2].

Clinical Implications

The use of LESIs as a chronic lumbar pain treatment should be integrated into a multimodal approach instead of being used as an independent treatment option. The short-term effects of LESIs on pain reduction and functional improvement in LSS and radiculopathy are regarded as their main objective [51,59]. Combining injections with physical rehabilitation, ergonomic modification, and psychological assistance provides the best results [52]. In addition, patient selection is also a determinant of patient outcomes. LESIs are primarily indicated for nonsurgical patients and those requiring temporary pain relief during the recovery process. Acute radiculopathy that presents with confirmatory imaging has been observed to respond optimally, whereas chronic axial pain or severe fixed stenosis is unlikely to respond [51]. The subtype of disc pathology must also be taken into account when choosing treatment methods and techniques, as well as unilateral, bilateral, or multilevel localization of pain. The choice of technique ought to be pathology-oriented. Transforaminal injection is performed directly on an affected nerve root, providing better relief in unilateral radiculopathy [51,52,66,67]. A bilateral or central stenosis is better suited to interlaminar injections [62-65]. Multi-level disease or post-laminectomy syndromes are most appropriate to caudal injections [53,56,60,61]. The issue of safety is also very important. Non-particulate steroids (e.g., dexamethasone) have now become the preferred method over particulate agents because they have lower embolic infarction and neurologic injury risks [68]. Unfortunately, chronic exposure to corticosteroids has been linked to bone loss, adrenal suppression, and systemic effects in vulnerable patient groups like postmenopausal women [69,70]. Hence, bone density and exposure to steroids per annum should be tracked and restricted in the high-risk groups. New protocols like image-guided precision targeting, adjunct use of hyaluronidase, and the combination therapies with PRP or non-steroidal injectates can be used to increase the effectiveness and reduce systemic exposure. Lastly, patient education is also critical. A clear and achievable expectation setting, including the focus on short-term relief and the need to adhere to harsher treatment regimes, can lead to reduced dissatisfaction and higher compliance with the overall treatment. As a bridge therapy, LESIs can provide rehabilitation, decrease the dependence on steroids, and postpone surgery with appropriate use.

Limitations and Future Directions

As it stands, LESIs are strongly supported by literature for their short-term benefits; however, studies reveal that study design limitations, small sample sizes, and variation in patient selection and outcomes make it difficult to generalize the conclusions on a broader level. Several studies report varying injection techniques, the type of steroid studied, and diagnostic criteria used, which makes it difficult to draw comparisons. Lastly, studies vary in the duration of follow-up of patient outcomes after six months, which hinders clear conclusions on the optimum number of injections or injection frequency.

Future research can address these issues by focusing on the standardization of protocols in RCTs and consistent longer follow-ups for the assessment of safety and efficacy. There is a significant need for more comparative studies on LESIs against alternative therapies such as minimally invasive surgery, radiofrequency ablation, and biologics. Perhaps there is also a role for the utility of LESI plus concurrent non-pharmacologic interventions versus LESI alone. Further research can further evaluate QALY in LESIs compared to traditional surgical interventions, as this area of research appears to be sparse. Another potential avenue of research is predictors of treatment response to help healthcare professionals in personalizing care and improving the quality of long-term results.

## Conclusions

LESIs are efficacious for short-term solutions of pain and disability associated with chronic lumbar radiculopathy and specific cases of spinal stenosis, particularly when adjusted according to the core pathology and delivered appropriately. Long-term benefits, however, are inconsistent, and there is often a need for repeated interventions, as would be expected considering the mechanism of action. Despite this controversy, LESIs are still considered to be generally safe and a minimally invasive option that helps avoid or reduce the requirement of surgical options. Additionally, evidence supports LESIs as being significantly more cost-effective than surgical interventions, and through QALY gained per intervention, ESI may be the most cost-effective method for controlling early-stage LSS. If used cautiously and adjusted in accordance with the patient factors and latest evidence, LESIs are a valuable asset in the toolkit for management of chronic lumbar spinal pain without surgery.
